# Supplementary material for: A yeast phenomic model for the influence of Warburg metabolism on genetic buffering of doxorubicin
Source: Cancer Metab. 2019 Oct 23;7:9. doi: 10.1186/s40170-019-0201-3 (PMC6806529; doi:10.1186/s40170-019-0201-3)
Supplement: Supplementary file 9 — Additional file 9. GO term-specific heatmaps for REMc/GTF-enriched clusters. GO term-specific heatmaps for significant GO process terms were generated as described in methods and Figs. 3 and 4. Any related child terms are presented in subsequent pages of the parent file name. GO terms with more than 100 children, with 2 or fewer genes annotated to the term, or a file size over 300KB are not shown. All heatmaps are generated with the same layout (see Figs. 3 and 4). [file 40170_2019_201_MOESM9_ESM.bz2 › Additional_File9_GOTermHeatmaps/Additional_File9_GOTermHeatmap/translational_initiation.pdf]

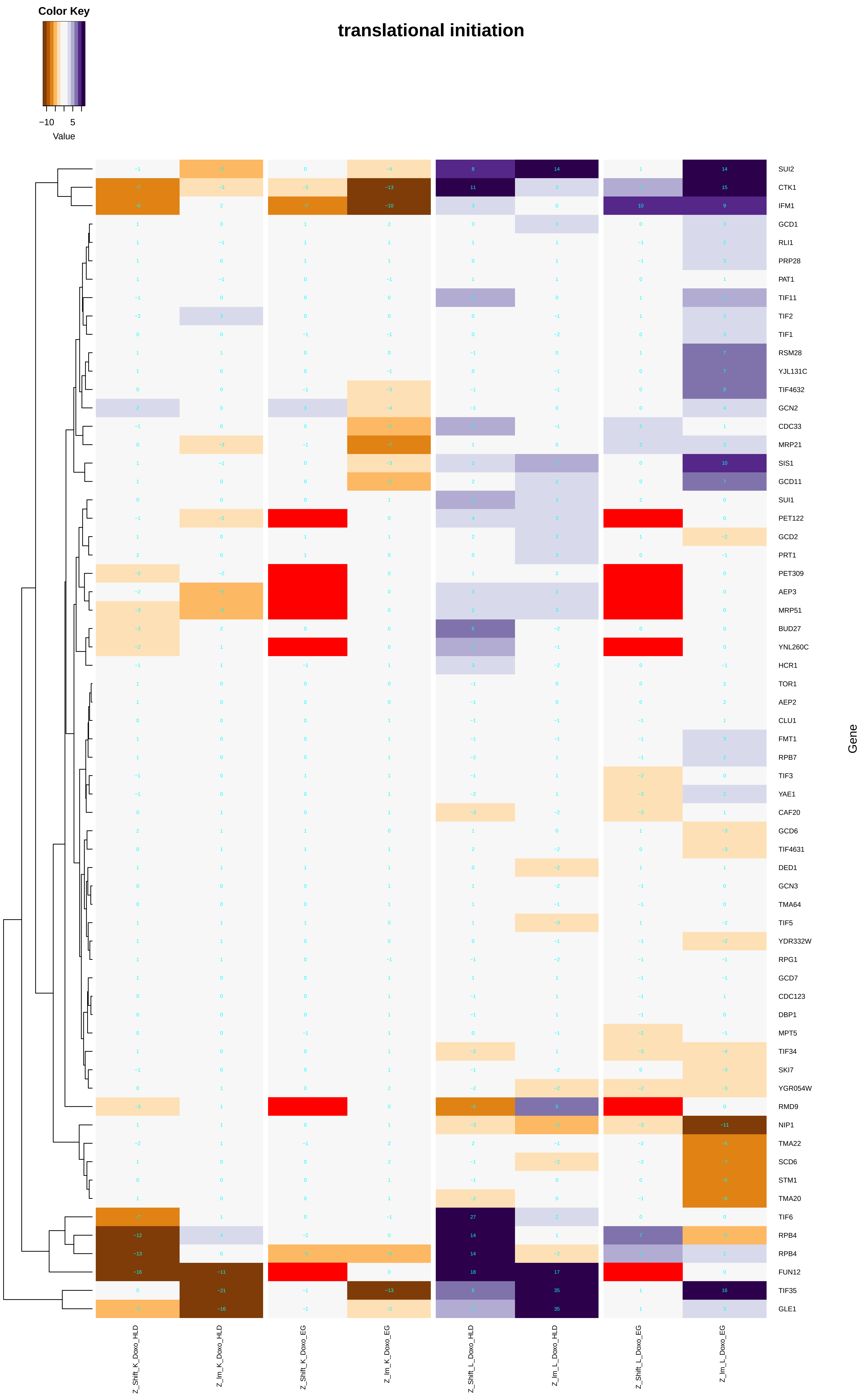
Color Key
−10 5
Value
translational initiation
SUI2
CTK1
IFM1
GCD1
RLI1
PRP28
PAT1
TIF11
TIF2
TIF1
RSM28
YJL131C
TIF4632
GCN2
CDC33
MRP21
SIS1
GCD11
SUI1
PET122
GCD2
PRT1
PET309
AEP3
MRP51
BUD27
YNL260C
HCR1
TOR1
AEP2
CLU1
FMT1
RPB7
TIF3
YAE1
CAF20
GCD6
TIF4631
DED1
GCN3
TMA64
TIF5
YDR332W
RPG1
GCD7
CDC123
DBP1
MPT5
TIF34
SKI7
YGR054W
RMD9
NIP1
TMA22
SCD6
STM1
TMA20
TIF6
RPB4
RPB4
FUN12
TIF35
GLE1
Gene
Z_Shift_K_Doxo_HLD
Z_Im_K_Doxo_HLD
Z_Shift_K_Doxo_EG
Z_Im_K_Doxo_EG
Z_Shift_L_Doxo_HLD
Z_Im_L_Doxo_HLD
Z_Shift_L_Doxo_EG
Z_Im_L_Doxo_EG

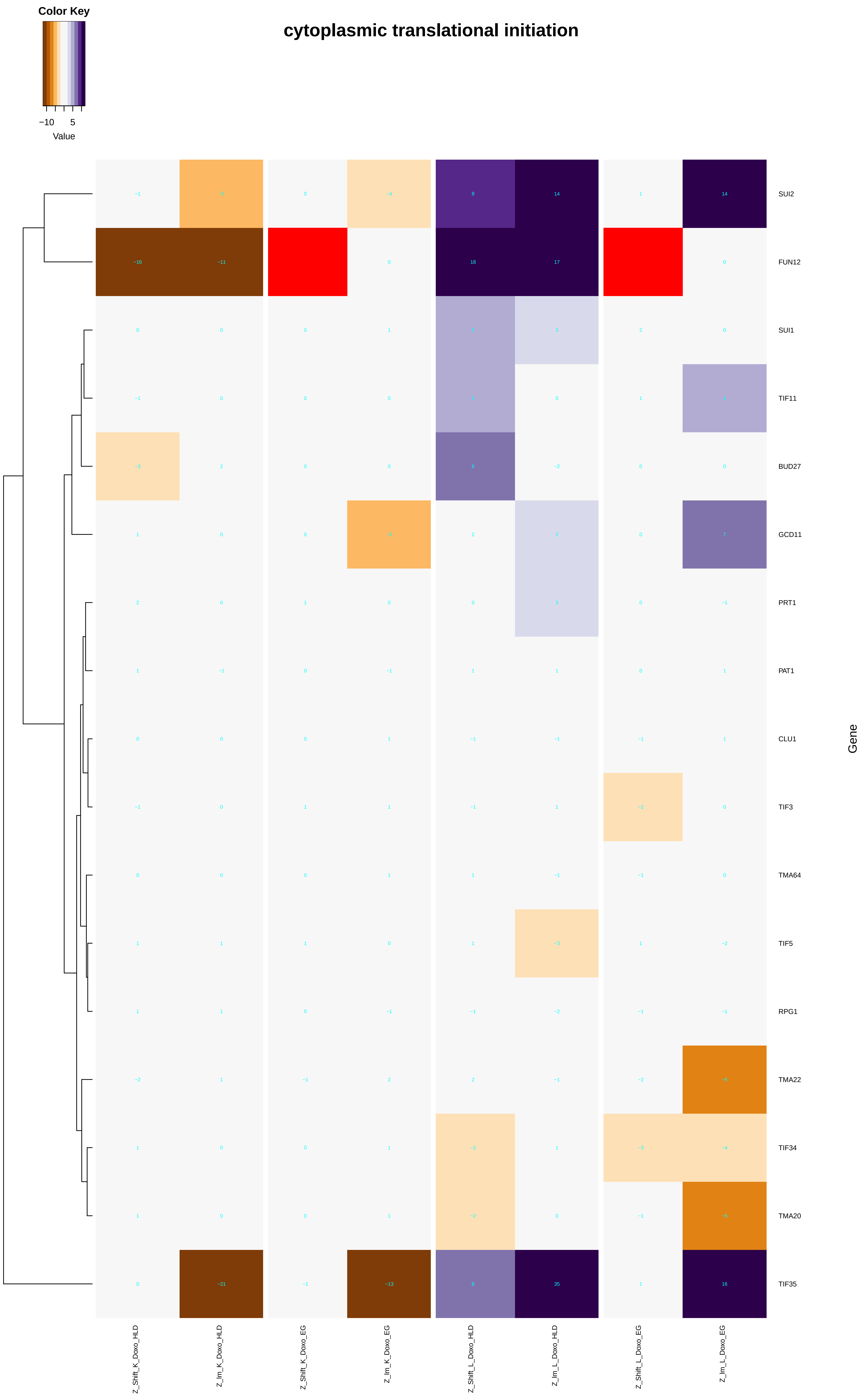

Color Key
−10
5
Value
cytoplasmic translational initiation
SUI2
FUN12
SUI1
TIF11
BUD27
GCD11
PRT1
PAT1
CLU1
TIF3
TMA64
TIF5
RPG1
TMA22
TIF34
TMA20
TIF35
Gene
Z_Shift_K_Doxo_HLD
Z_Im_K_Doxo_HLD
Z_Shift_K_Doxo_EG
Z_Im_K_Doxo_EG
Z_Shift_L_Doxo_HLD
Z_Im_L_Doxo_HLD
Z_Shift_L_Doxo_EG
Z_Im_L_Doxo_EG

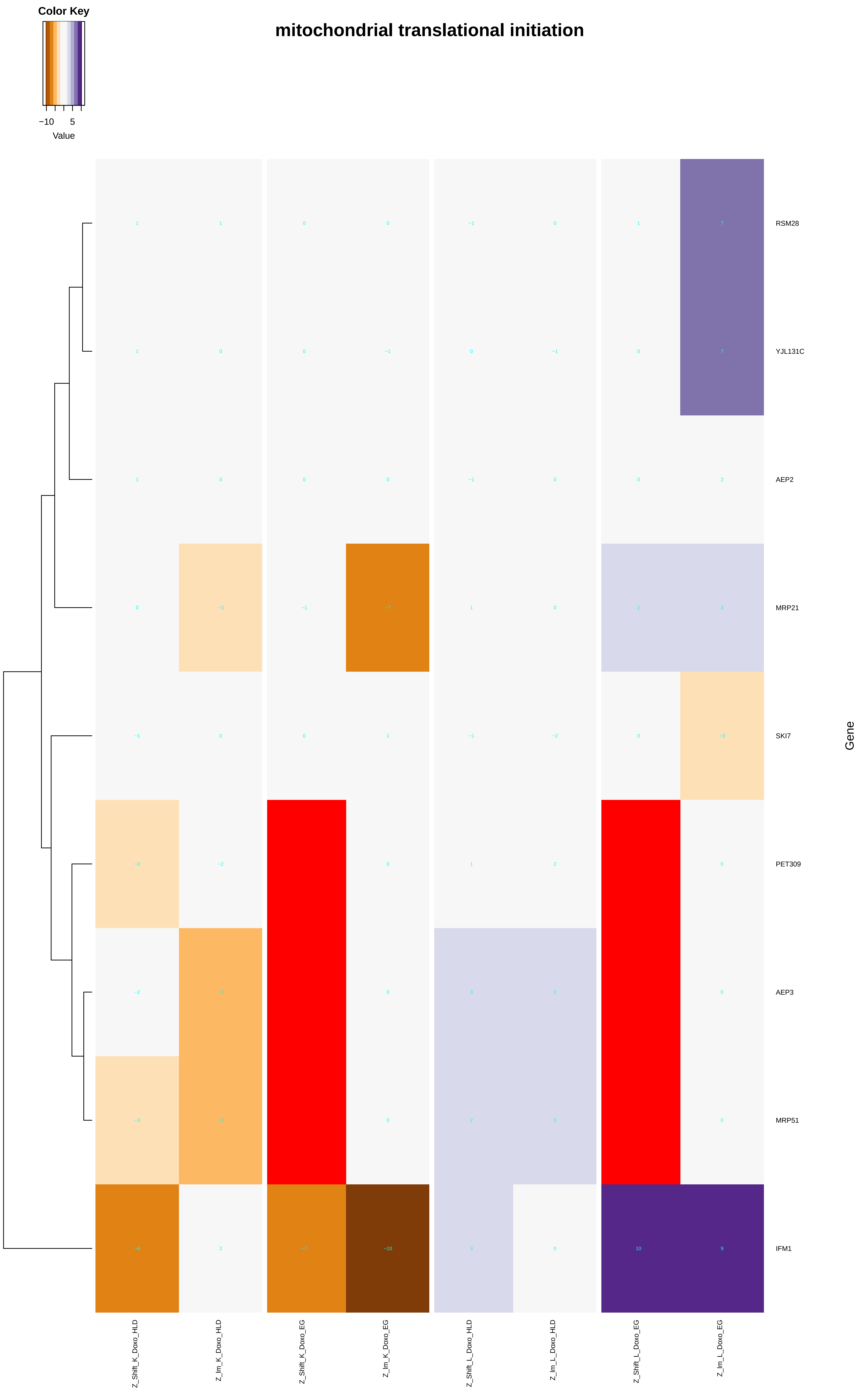
Color Key
−10
5
Value
mitochondrial translational initiation
RSM28
YJL131C
AEP2
MRP21
SKI7
PET309
AEP3
MRP51
IFM1
Gene
Z_Shift_K_Doxo_HLD
Z_Im_K_Doxo_HLD
Z_Shift_K_Doxo_EG
Z_Im_K_Doxo_EG
Z_Shift_L_Doxo_HLD
Z_Im_L_Doxo_HLD
Z_Shift_L_Doxo_EG
Z_Im_L_Doxo_EG

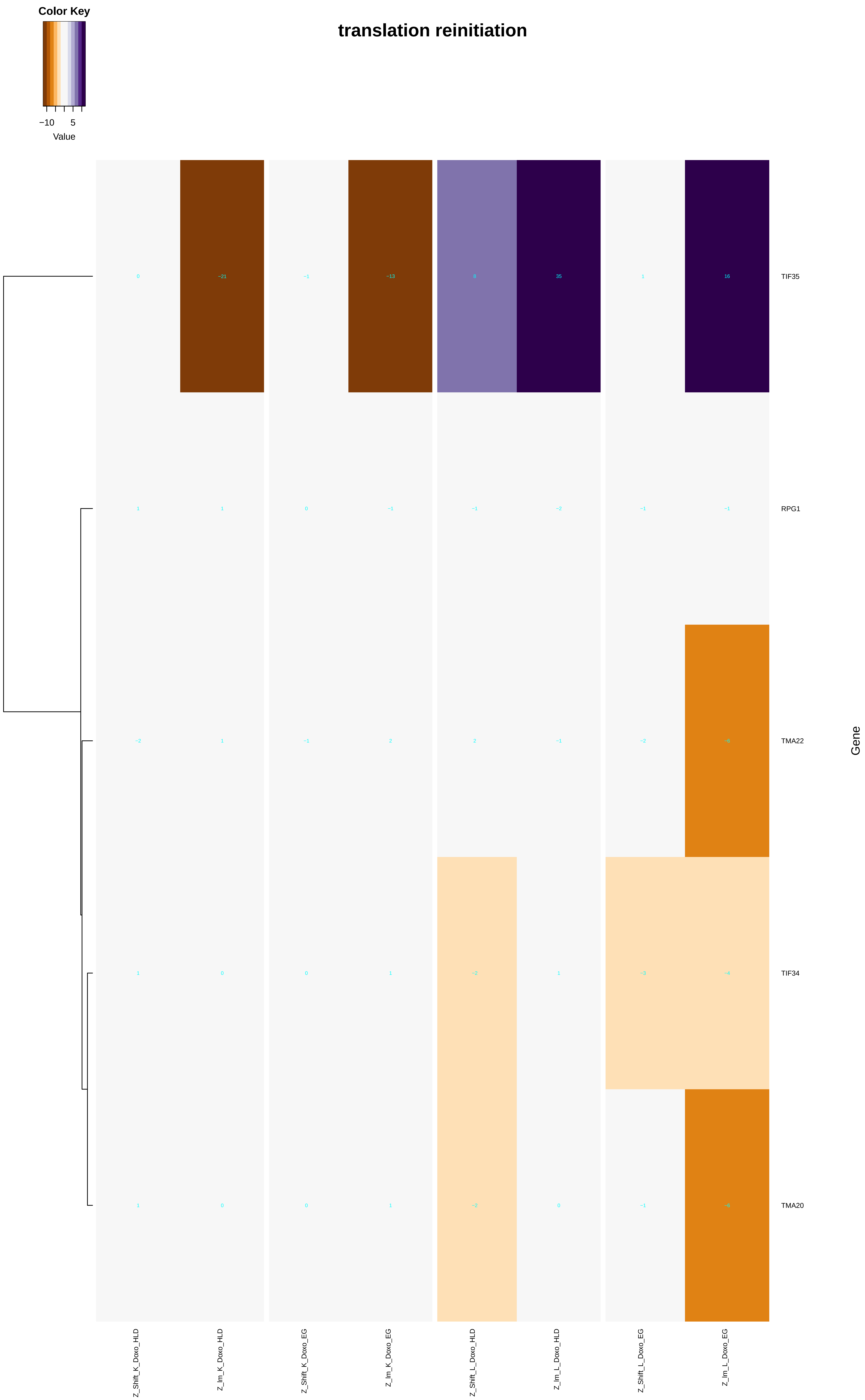
translation reinitiation
Color Key
−10
5
Value
TIF35
RPG1
TMA22
TIF34
TMA20
Gene
Z_Shift_K_Doxo_HLD
Z_Im_K_Doxo_HLD
Z_Shift_K_Doxo_EG
Z_Im_K_Doxo_EG
Z_Shift_L_Doxo_HLD
Z_Im_L_Doxo_HLD
Z_Shift_L_Doxo_EG
Z_Im_L_Doxo_EG
